# Effective preparation of a monoclonal antibody against human chromogranin A for immunohistochemical diagnosis

**DOI:** 10.1186/s12896-018-0436-z

**Published:** 2018-05-04

**Authors:** Danping Zhang, Chengjie Xie, Rongzhi Wang, Qinghai Yang, Huiling Chen, Sumei Ling, Shihua Wang, Kunzhi Jia

**Affiliations:** 1 Key Laboratory of Pathogenic Fungi and Mycotoxins of Fujian Province, Key Laboratory of Biopesticide and Chemical Biology of Education Ministry, and School of Life Sciences, Fujian Agriculture and Forestry University, Fuzhou, 350002 China; 2 Fuzhou Maixin Biotech. Co., Ltd, Fuzhou, 350100 China

**Keywords:** Chromogranin A, Monoclonal antibody, Immunohistochemistry, Specifility, iELISA

## Abstract

**Background:**

Human chromogranin A (CgA) is a ~ 49 kDa secreted protein mainly from neuroendocrine cells and endocrine cells. The CgA values in the diagnosis of tumor, and in the potential role in prognostic and predictive tumor as a biomarker.

**Results:**

The synthesized gene of *CgA* coding area was cloned and expressed as fusion protein CgA-His in procaryotic system. Then the purified CgA-His protein was mixed with QuickAntibody-Mouse5W adjuvant, and injected into mice. The CgA-His protein was also used as coating antigen to determine the antiserum titer. By screening, a stable cell line named 4E5, which can generate anti-CgA monoclonal antibody (mAb), was obtained. The isotype of 4E5 mAb was IgG_2b_, and the chromosome number was 102 ± 4. Anti-CgA mAb was purified from ascites fluid, and the affinity constant reached 9.23 × 10^9^ L/mol. Furthermore, the specificity of the mAb was determined with ELISA, western blot and immunohistochemistry. Results indicated that the mAb 4E5 was able to detect chromogranin A specifically and sensitively.

**Conclusions:**

A sensitive and reliable method was successfully developed for rapid production of anti-CgA mAb for immunohistochemistry diagnosis in this study, and the current study also provides conclusive guidelines for preparation of mAbs and implements in immunohistochemistry diagnosis.

## Background

Human chromogranin A (CgA) consists of 457 amino acid with molecular weight ~ 49 kDa, and its gene is located in chromosome 14 of *Homo sapiens*. As a member of Granin protein family, CgA is mainly produced by endocrine cells or neuroendocrine cells and presents in the secretory dense core granules of neuroendocrine tissues [1, 2]. These CgA secrecting tissues include small intestine, large intestine, adrenal medulla and pancreatic islets [1, 2].

Further studies found that CgA was highly expressed in some tumors, and then determined as excellent marker in carcinoid tumor, pheochromocytomas, and paragangliomas [1, 3–5]. Compared with other neuroendocrine markers, CgA has the higher specificity for the detection of neuroendocrine tumors. Thus the CgA was widely used in immunohistochemistry (IHC) to diagnose a benign or malignant tumor, and determine the tumor grade. In this sense, it values to develop sensitive monoclonal antibodies (mAb) against CgA for IHC diagnosis.

Compared with the time-consuming and technique-requiring method to express CgA in a eukaryotic cell line, a method for CgA expression in prokaryotic system was developed. Hence, a cost-effective, feasible and reliable method was established to prepare an anti-CgA mAb by expressing CgA in *E. coli*. The prepared mAb against CgA from prokaryotic system can be used in Western Blot and IHC diagnosis based on its high specificity in this study.

## Methods

### Materials and chemicals

The anti-6 × His tag monoclonal antibody and HRP-labeled goat anti-mouse IgG were purchased from ZSGB-Bio Co., Ltd. (Beijing, China). The cocktail of LK2H10 and PHE5 antibodies, and MaxVisionTM/HRP IHC kit were provided by Fuzhou Maixin Biotech Co., Ltd. (Fuzhou, China). SP12 antibody was from Thermo Fisher Scientific (Shanghai, China). Hypersensitivity ECL chemiluminescence detection kit was purchased from Wuhan Sanying Co., Ltd. (Wuhan, China). Myeloma cells SP2/0 were stored in our laboratory. Hypoxanthine, aminopterin and thymidine supplement (HAT), hypoxanthine and thymidine supplement (HT) and polyethylene glycol 1450 solution (PEG 1450) were purchased from Sigma-Aldrich (Shanghai, China). RPMI medium 1640 powder and fetal bovine serum (FBS) were purchased from Thermo Fisher (Shanghai, China). Balb/c mice were from Wushi Animal Laboratory (Shanghai, China). QuickAntibody-Mouse5W adjuvant (Quickadjuvant) was purchased from Beijing Biodragon Immunotechnologies Co., Ltd. (Beijing, China). All animal experiments obeyed the protocols approved by the Animal Ethics Committee of the Fujian Agriculture and Forestry University.

### Cells cultivation

Murine myeloma cell line SP2/0 stored in our laboratory were cultured in RPMI-1640 medium with 10% FBS. Hybridoma cells were cultured in RPMI-1640 medium with HAT containing 20% FBS. After 10 d of cell fusion, hybridoma cells were cultured in RPMI-1640 medium with HT containing 20% FBS. A week later, the hybridoma cells were cultured in RPMI-1640 medium with 10% FBS.

### Codon optimization and synthesis of *CgA* gene

The identification of *CgA* is P10645 from the Uniprot, according to the database. The coding part of *CgA* was selected to optimize codon for expression in *E. coli* BL21 (DE3) and evaluated by graphical codon usage analyser (http://gcua.schoedl.de) [6]. The optimized DNA of *CgA* was synthesized by Nanjing Genscript Biotech Co., Ltd. (Nanjing, China).

### Expression and identification of the CgA fusion protein

The target gene was amplificated by PCR and digested by *EcoR*I/*Hind*III, then inserted into the plasmid pET-28a to transform *E.coli* BL21 (DE3) competent cells by calcium chloride transformation. Furthermore, the positive clone was induced by isopropyl-β-d-thiogalactoside (IPTG), and the target proteins were expressed and purified by affinity chromatography purification. Finally, the concentrations of CgA-His fusion protein were detected by BCA methods and Nanodrop (Thermofisher) [7]. The recombinant CgA protein was verified by western blot [8].

### Animal immunization and titer analysis by iELISA

The 6–8 weeks old mice (female) were injected intramuscularly in hind legs. The first immunization contained 60 μg CgA-His fusion protein mixed with 150 μL Quickadjuvant and 0.9% saline solution. Three weeks later, the mice were immunized once again. After a week of the third immunization, blood was abstracted from tail of mouse and tested by iELISA [9]. CgA-His protein as coating antigen was diluted by carbonate buffer (pH 9.6) to the concentration of 5 μg**/**mL. After coating, the plates were washed 3 times with 1 × PBS, and then blocked with 200 μL/well 5% PBSM at 37 °C for 2 h. Primary antibody (anti-CgA antiserum) was succession diluted in PBSM and incubated at 37 °C for 1 h. After washing, HRP-labeled goat anti-mouse IgG (1:8000 diluted by 5% PBSM, 100 μL/well) was added and incubated at 37 °C for 1 h. Plated was cleaned and 100 μL substrate solutions were added into per well with incubation at 37 °C for 10 min. Finally, the reaction was stopped by 2 M H_2_SO_4_ (50 μL per well), and the titer was detected by micro-plate Reader under the optical density (OD) value at 450 nm [10, 11].

### Cell fusion and hybridoma screening

The mouse that had higher titer was injected intraperitoneally as the further immunization with 20 μg His-CgA fusion protein mixed with 100 μL 0.9% saline solution. Three days later, splenocytes were isolated from the immunized mouse and mixed with murine SP2/0 myeloma cells at a ratio of 10: 1 with the chemical reagent PEG1450, then distributed into 96-well micro-titer plates in which feeder cells had been added [10]. These cells were cultured in RPMI 1640 with 20% FBS/HAT medium at 37 °C and 5% CO_2_ incubator. Five days later, the 50% medium in micro-titer plates was substituted with the fresh one. Ten days later, the positive hybridoma cells were determined by iELISA. The positive clone with high titer was chosen for subclone, until the positive percentage was up to 100% [10, 11].

### Characterization of the positive hybridoma cells against CgA

Determination of the mAb isotype was identified according to the instruction of mouse Monoclonal Antibody Isotyping (IgA, IgM, IgG_1_, IgG_2a_, IgG_2b_, IgG_3_) kit [12]. Chromosome analysis was identified according to the publication in our lab [13]. The Hybridoma cells were stained by Giemsa solution, and the chromosome number was counted under the flurescence microscope.

### Production of anti-CgA mAb

A Balb/c mouse was injected intraperitoneally with 0.5 mL paraffin oil. Seven days later, approximately 1 × 10^6^ postive hybridoma cells were injected into the mouse abdominal cavity. After 1 week, the ascites fluid was collected by the needle and centrifuged at 12000 r/min for 20 min. The supernatant was absorbed and stored in − 20 °C fridge. According to the protocol of Protein G, the mAb was purified and analyzed by 10% SDS-PAGE [14]. The concentration of the purified mAb was determined by the BCA Protein Assay.

### Affinity determination of mAb

The affinity determination of monoclonal antibody against CgA was carried out by the publication [15]. The CgA-His protein (100, 50, 25, and 12.5 ng/mL for 4E5 mAb or 2.5, 1.5, and 1 μg/mL for LK2H10 and PHE5) was coated and the following steps are consistent with the protocol of iELISA above. The curve of diagram showed the relationship between the concentration of antibody as the abscissa and the value of absorption as the ordinate. Relative affinity of anti-CgA mAb was measured by determining the 50% inhibition of control values (IC_50_). And the affinity constant (K_aff_) was calculated by using the reported method [16].

### IHC tests of mAb

After being deparaffinized in xylene and rehydrated in graded alcohol, the formalin-fixed paraffin-embedded specimens were treated with boiling 10 mmol/L citrate buffer (pH 6.0) for 10 min using pressure cooker. After that samples were cooled to room temperature, washed 3 times with PBS, then blocked by 3% H_2_O_2_ for 10 min and washed by PBS again. Anti-CgA mAb (1:1000 for 4E5, LK2H10 and PHE5 or 1:200 for SP12) was added into the section and incubated at room temperature for 1 h. After final washing, MaxVisionTM/HRP-Polymer anti-mouse or anti-rabbit (for SP12) IHC kit - was dropped into the slice and incubated for 25 min, and then washed. The DAB chromogenic liquid was added into the section for 10 min, and then stopped with water. Then, the section was redyed with hematoxylin for 25 s and further stained for 30 s. The slice was dehydrated by a serial of concentrations of ethyl alcohol, and treated by xylene for 3 min. Finally, the section was sealed by neutral gum and observed under microscope.

### Determination of specificity of mAb

The specificity of mAb against CgA was determined by three kinds of methods. The first one is the iELISA. Different kinds of human proteins (B-cell lymphoma 6 protein-His (BCL6-His), programmed cell death 1 ligand 1 (PD1-L1), P-glycoprotein (PGY) and podoplanin-His (PDPN)) were generated and purified with the same methods as CgA protein using in this study. Human proteins as coating antigens were diluted by carbonate buffer (10 μg/mL) and added into the micro-titer plates (100 μL/well), respectively. And the negative control was added 100 μL/well carbonate buffer. Then, the following steps are consistent with the protocol of ELISA. The second method for identifying the specificity of the mAb is western blot. CgA-His protein was electrophoresed in 10% SDS-PAGE gel, transferred into the PVDF membrane, and blocked with 5% PBSM. The anti-CgA mAb as primary antibody was diluted 1:8000 with 5% PBSM to incubate with the PVDF membrane for 1 h, and the following steps was the same to ELISA. Finally ECL chemiluminescence detection reagents were added, and the pictures were taken by fully functional multicolor fluorescence imaging instrument. The third method is IHC method, which consistent with the experiment of IHC test above. After the section from small cell lung carcinomas, islet cell carcinoma and adrenal gland tissues were obtained, all of them were dropped of the anti-CgA mAb with dilution, and MaxVisionTM/HRP reagent and the DAB chromogenic liquid were added subsequently. These sections were dealt with a serial of chemicals followed by observation [10, 11].

#### Determination of 4E5 mAb epitope

The optimized mature CgA gene was divided into four gene fragments. Each CgA gene fragment contains 110 amino acid coding codons except the fourth fragment, which has 109 amino acid coding codons. CgA gene fragments were synthesized by General Biosystems (Chuzhou, China) and cloned into pET-28a plasmid at *BamHI*/*Hind*III site. These fragments fused with His tags were expressed in *E.coli* BL21 (DE3). The protein expression was analyzed with SDS-PAGE, and the epitope of 4E5 was determined with western blot. The SDS-PAGE and immunoblotting steps are following the protocols described above.

## Results

### Cloning and expression of the *CgA* in prokaryotic system

**Fig. 1:**
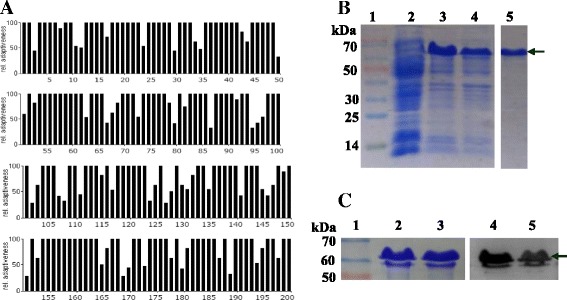
Cloning and Expression of CgA in prokaryotic system. **a** Identification of the optimized sequence by graphical codon usage analyser (http://gcua.schoedl.de). **b** Purification and identification of CgA-His. Lane 1: Marker (Blue plus II protein marker), lane 2: the total protein of empty vector pET-28a, lane 3–4: the total protein of pET-28a-CgA in Transetta (DE3), lane 5: the purified CgA-His protein. **c** Western blot result of CgA-His. Lane 1: Marker (Blue plus II protein marker), lane 2–3: the duplicate for purified CgA-His protein, lane 4–5: the duplicate for western blot result of CgA-His reacted with anti-His_6_ tag monoclonal antibody

To clone human *CgA* gene in prokaryotic system, the sequence of mature *CgA* was synthesized after codon optimization for *E. coli* expression. According to analysis by the graphical codon usage analyser (http://gcua.schoedl.de), the results showed that the relative adaptivenesses of codons were range from 20% to 100%, indicating that the codons could be well expressed in *E. coli* BL21 (DE3) (Fig. 1a). After verification, *CgA* was successfully cloned into the vector pET-28a, then expressed in *E. coli* BL21 (DE_3_) (Fig. 1b). The expressed *CgA* was then purified with affinity method, and the result in Fig. 1b showed that the specific and distinct target bands were observed at 60~ 70 kD (CgA-His). The expressed and purified CgA protein was confirmed with western blot analysis (Fig. 1c), indicating that CgA was successful expressed and purified in prokaryotic system.

### Mice immunization and screening of positive hybridoma clones

**Fig. 2:**
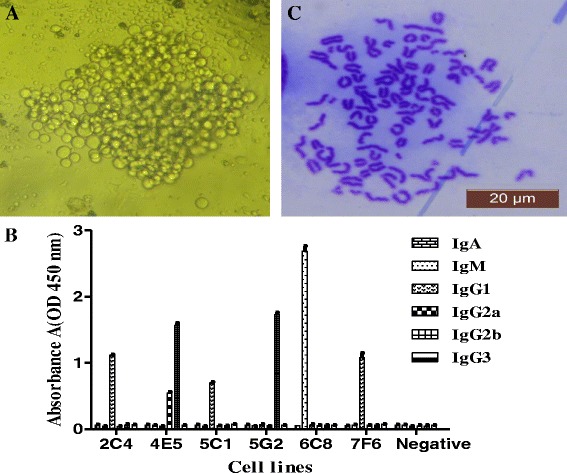
Screening of positive hybridoma clones. **a** The observation of hybridoma cells cultured in HAT medium. Hybridoma cells were observed after 6 d of fusion (× 100). **b** The isotype result of positive hybridoma cells. The isotype of monoclonal antibody was determined by isotyping (IgG1, IgG2a, IgG2b, IgG3, IgA, IgM) kit, and the isotype of 4E5 is IgG2b. **c** Chromosome analysis of 4E5 hybridoma cell (10 × 100). The chromosome number of 4E5 cell line is 102 ± 6

After four times immunization in Balb/c mice with purified CgA, the titer of antiserum of the immunized mice was determined by iELISA. The result showed that the titer of mice immunized with CgA-His protein was significantly higher than that of control mice, which indicated that CgA-His protein successfully stimulated the production of antibody against CgA. The mouse No.1 which has high serum titer against anti-CgA was used for cell fusion. Spleen cells from the mouse No.1 were isolated and fused to SP2/0 myeloma cell by PEG1450 at the ratio of 10: 1. After cell hybrization, the scale of clones reached 70% bottom of 96-well micro-titer plate after 6 day of fusion (Fig. 2a). Six hybridoma cell lines stably secreting anti-CgA antibody were screened out, and named 2C4, 4E5, 5C1, 5G2, 6C8 and 7F6, respectively. The isotype of the anti-CgA mAb was determined with isotyping mAb kit, and the results showed that the isotype of monoclonal antibody 2C4, 4E5, 5C1, 5G2, 6C8 and 7F6 are IgG1, IgG2b, IgG1, IgG2b, IgM and IgG3, respectively (Fig. 2b). The cell line of 4E5 showed a good repeats in ELISA, Western blot and IHC, therefore 4E5 was chosen for the further experiments. The chromosome number of 4E5 cell line is 102 ± 6 (Fig. 2c), suggesting that the chromosome number of the hybridoma cell 4E5 was approximately equal to the total chromosome number of spleen cell (39 ± 1) and myeloma cell SP2/0 (66 ± 4). The results further demonstrated that the 4E5 cell line was successfully fused.

### Purificaion of the anti-CgA mAb

**Fig. 3:**
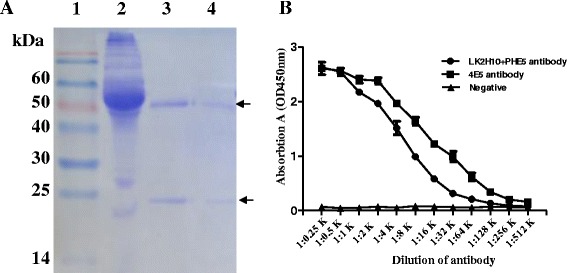
Purification of the anti-CgA mAb. **a** SDS-PAGE analysis of the purified mAb. Lane 1: Marker (Precision Plus Protein Dual color standards), lane 2: ascites fluid, lane 3–4: the duplicate purified mAb against CgA. **b** Titers of the purified 4E5 anti-CgA mAb and control antibody were determined by iELISA

To get enough CgA mAb, the 4E5 hybridoma cells were injected into the peritoneal cavity of pristine-primed Balb/c mice. Then the mice ascites fluids were collected and then purified with Protein G. As shown in Fig. 3a, two distinct bands appeared and miscellaneous band lacked after purification, consistent with the heavy chain and light chain of antibodies. The 50 kDa heavy chain and the 25 kDa light chain were observed in lane 3 and lane 4, indicating that 4E5 mAb was successfully purified by Protein G (Fig. 3a). The final concentration of the purified 4E5 mAb was 0.13 mg/mL determined with BCA protein assay, and the titer of anti-CgA mAb reached up to 5.12 × 10^5^ with iELISA method (Fig. 3b). We also compared the titer of 4E5 mAb with that of LK2H10 and PHE5, a widely used anti-CgA mixture [17, 18]. Results showed that the titer of 4E5 is higher than that of anti-CgA mixture (Fig. 3b), demonstrating that the anti-CgA 4E5 mAb we purified was suitable for further characterization.

### Characterization of the anti-CgA mAb

**Fig. 4:**
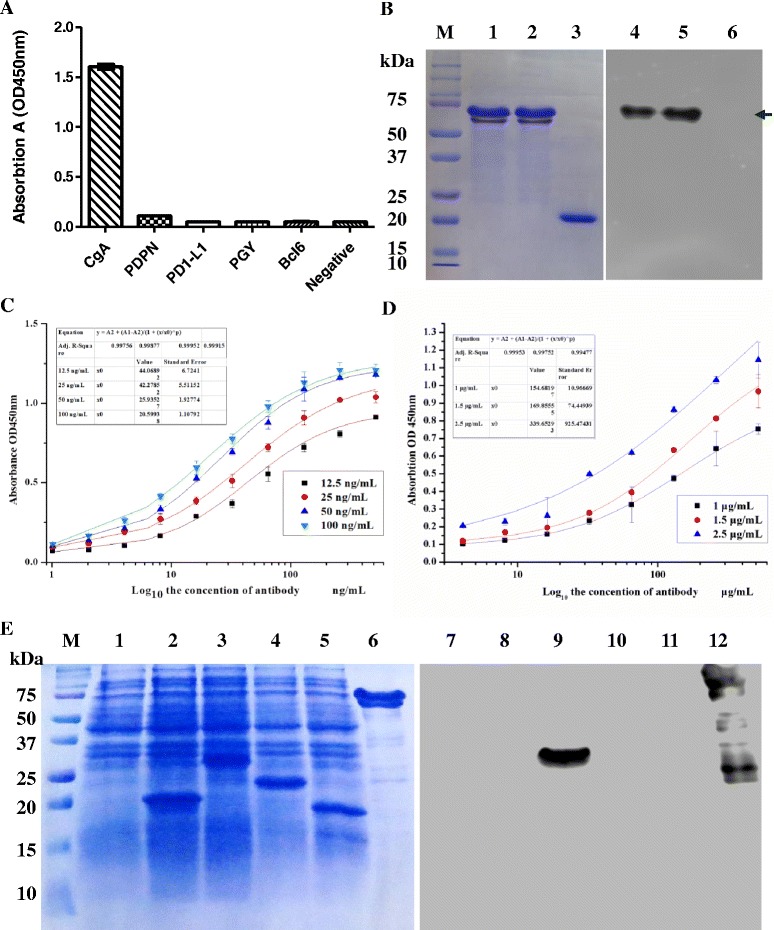
Characterization of the purified 4E5 mAb. **a** The specificity of the 4E5 mAb was determined by indirect ELISA (BCL6: B-cell lymphoma 6 protein, PD1-L1: Programmed cell death 1 ligand 1, PDPN: Podoplanin, PGY: P-glycoprotein). **b** The specificity of the 4E5 mAb was determined by Western Blot. Lane M: Marker, Lane 1–2: His-CgA protein duplicates, Lane 3: Random control (His-PD1-L1 human protein), Lane 4–6: The result of Western Blot. The proteins in Lane 4 and 5 are his-CgA protein duplicates, and Lane 6 was random control (His-PD1-L1). **c** Affinity of 4E5 mAb was analyzed by iELISA. Different concentrations (12.5, 25, 50, and 100 ng/mL) of coating antigen (CgA-His) were used to determine the affinity constant which is 9.23 × 10^9^ L/mol. **d** Affinity of control antibodies (LK2H10 and PHE5) was analyzed by iELISA. Different concentrations (1, 1.5, and 2.5 μg/mL) of coating antigen (CgA-His) were used to determine the affinity constant which is 3.89 × 10^8^ L/mol. **e** Epitope of 4E5 mAb was determined with western blot. Lane1–7: the result of SDS-PAGE analysis. Lane M: Marker, lane 1: the total protein of BL21 (DE3) with empty vector pET-28a, lane 2: the total protein of BL21 (DE3) with pET-28a-CgA fragment 1 (F1), lane 3: CgA fragment 2 (F2), lane 4: CgA fragment 3 (F3), lane 5: CgA fragment 4 (F4), lane 6: CgA. Lane 7–12: The result of Western Blot. Lane 7: the total protein of BL21 (DE3) with empty vector pET-28a, lane 8: CgA fragment 1 (F1), lane 9: CgA fragment 2 (F2), lane 10: CgA fragment 3 (F3), lane 11: CgA fragment 4 (F4), lane 12: CgA

To assay the value of anti-CgA 4E5 mAb, the affinity and specificity of antibodies were determined by iELISA method [10, 11]. The results showed that the 4E5 mAb was sensitive in response to CgA-His, and not cross-reactive to random protein control, such as PDPN, PD1-L1, PGY, and BCL6. (Fig. 4a), indicating the 4E5 antibodies had high specificity for CgA. The specifity of 4E5 antibodies were also evaluated with Western Blot. As shown in Fig. 4b, the 4E5 mAb specifically recognized the target proteins CgA-His rather than control antigens. The affinity analysis of 4E5 mAb was also performed with iELISA, and the results showed that the affinity of 4E5 mAb was up to 9.23 × 10^9^ L/mol (Fig. 4c). In contrast, the affinity of anti-CgA mixture (LK2H10 and PHE5) was similarly determined as 3.89 × 10^8^ L/mol (Fig. 4d). These results indicated that the 4E5 mAb was more sensitive to CgA than LK2H10 and PHE5. Considering the mixture of LK2H10 and PHE5 antibodies was widely used in CgA detection [17, 18], thus the 4E5 mAb was value for further application for CgA detection or diagnosis. To determine the epitope of 4E5 antibody, we divided CgA gene into four fragments and expressed these gene fragments (Fig. 4e). Each fragment has about 110 amino acids. As shown in Fig. 4e, the second protein fragment (CgA_111–220_) was easily recognized by 4E5 antibody as CgA protein with WB, demonstrating the CgA_111–220_ fragment was the epitope of 4E5 antibody.

### Tissue section detection for the mAb against CgA

**Fig. 5:**
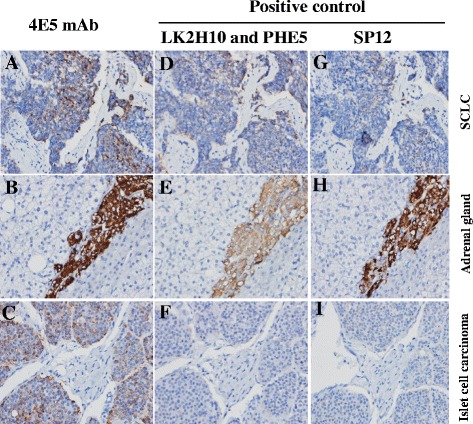
The immunohistochemistry tests of mAb in difference tissues. The immunohistochemistry tests of 4E5 mAb was determined in small cell lung carcinomas (SCLC) (**a**) (× 200), adrenal gland tissues (**b**) (× 400), and Islet cell carcinoma (**c**) (× 200). The images of (**d**, **e** and **f**) showed the staining of LK2H10 and PHE5 mouse antibodies for corresponding tissues, as positive control. The images of (**g**, **h** and **i**) showed the staining of SP12 rabbit antibody for corresponding tissues, as positive control

Considering the CgA detection in clinic diagnosis, the 4E5 antibodies were tested with IHC assay. Different tissue samples were detected with Anti-CgA 4E5. As shown in Fig. 5, the 4E5 mAb is able to sensitively detect CgA equal to the positive control group (LK2H10 and PHE5 mixture and SP12). Thus 4E5 mAb can be used to specifically recognize CgA in the small cell lung carcinomas (SCLC), adrenal gland tissues, and Islet cell carcinoma (Fig. 5). The stained cells indicated tumor cells or CgA secreting cells were located in these tissues, which were valuable in clinical practice. In addition, the staining degree in 4E5 group was much deeper than the positive group, suggesting that the sensitivity of the 4E5 mAb was better than LK2H10 and PHE5 or SP12 antibodies. All these results showed the 4E5 mAb had good potential value to detect CgA secreting tumor cells on clinic.

## Discussion

In this study, we used a prokaryotic system to express CgA antigen. We firstly optimized codons of *CgA* gene for *E. coli* expression. Then we successfully cloned the CgA gene in prokaryotic expressing vector. Compared with CgA expression in eukaryotic system, CgA was easily and fast acquired by using *E. coli* host cell to construct the high level prokaryotic expressing system. Thus time and cost were saved by using the prokaryotic expression system. However, the CgA was not well expressed in *E. coli* with the eukaryote codon bias according to our attempt. Our codons optimization of CgA successfully resolved the problem of low CgA expression in prokaryotic system. Thus, an effective and reliable method has been established to prepare the anti-CgA mAb by expressing CgA in *E. coli* using the optimized codons*.*

Meanwhile, our results indicated that a valuable antibodies secrecting hybridoma for CgA was successfully screened with the antigen from prokaryotic system. As shown in Fig. 4a, the 4E5 mAb from hybridoma specifically reacted with CgA rather than other control antigens (PDPN, PD1-L1, PGY, BCL6) with iELISA method. The 4E5 mAb specifically for CgA was further confirmed with WB analysis (Fig. 5). Our results also showed that the affinity of 4E5 mAb was high up to 9.23 × 10^9^ L/mol (Fig. 4c). This affinity is far beyond the baseline (10^7^ L/mol) required for further applications [19], suggesting that the 4E5 mAb has good potential value in clinic application.

Moreover, although eukaryotic proteins usually underwent various post-translational modifications, CgA specific antibody applicable in IHC has been successfully generated with antigen from prokaryotic system in this study. We further characterized the epitope of the CgA specific antibody (Fig. 4e). To our knowledge, this epitope is little reported previously. This epitope should be valuable for the generation of CgA specific IHC antibodies. The 4E5 antibodies recognize this epitope, CgA_111–220_, will be useful for the detection of full-length CgA or long fragment of CgA, considering CgA_110–220_ rarely included in polypeptides from CgA [20]. Interestingly, CgA_111–220_ consist of many charged amino acids expresses 10–20 kDa lag in electrophoresis (Fig. 4e), leading to the lag effect of full-length CgA in electrophoresis compared with the theory molecular weight.

Our study also showed that the 4E5 mA is qualified for clinic application with IHC experiments. Nowadays, the cocktail of LK2H10 and PHE5 antibodies against CgA has been implemented in IHC diagnosis [17, 18, 21]. Thus it is used as the positive control in measuring the application of 4E5 mAb in clinic IHC experiments. We also used a CgA specific rabbit mAb, SP12, as positive control, which is also accepted as CgA detection [22, 23]. Our results showed the effect of 4E5 mAb can even better detect CgA in clinic samples than the cocktail of LK2H10 and PHE5 and SP12 antibody (Fig. 5), suggesting that it was valuable in further clinic application. The superiority of 4E5 mAb in CgA detection by immunohistochemistry may be mainly due to the high titer and affinity of 4E5 mAb compared with those of control group, which is observed in the example of comparison between 4E5 mAb and the LK2H10 and PHE5 antibodies (Figs. 3b, 4c, and d). As to the control antibodies, although there are other CgA-specific antibodies applicable in IHC, such as rabbit polyclonal anti-CgA antibody and EP38 rabbit mAb [24, 25], our positive controls (LK2H10&PHE5 and SP12) including mouse and rabbit monoclonal antibodies are still representative. On the other hand, although our results were shown just from lung carcinomas, adrenal gland and Islet cell carcinoma, it is reasonable that the 4E5 is also applicable in other related tissues.

## Conclusions

In summary, we have successfully cloned and expressed CgA antigen with an effective and reliable method. This CgA antigen from prokaryotic system is obviously suitable for preparing a Chromogranin A mAb. Moreover, a high specific and affinity antibody named 4E5 for CgA has been screened in our study. The 4E5 mAb against CgA was valuable in IHC diagnosis. Our study provided an alternative method for the rapid preparation of CgA mAb for clinic application, considering the potentially complicated regulation and presence of CgA in various diseases.

## Abbreviations

BCL6: B-cell lymphoma 6 protein
CgA: Chromogranin A
iELISA: Indirect Enzyme Linked Immunosorbent Assay
IHC: Immunohistochemistry
IPTG: Isopropyl-β-d-thiogalactoside
PD1-L1: Programmed cell death 1 ligand 1
PDPN: Podoplanin
PGY: P-glycoprotein; small cell lung carcinomas (SCLC)
